# Validation and measurement properties of the Male and Female Fertility Knowledge Inventories (MFKI and FFKI) in Iranian couples

**DOI:** 10.1186/s12978-024-01795-0

**Published:** 2024-04-23

**Authors:** Sepideh Mashayekh-Amiri, Mohammad Asghari Jafarabadi, Behnam Molaie, Fatemeh Rashidi, Elnaz Hemati, Fatemeh Aliasghari, Mojgan Mirghafourvand

**Affiliations:** 1 Student of Midwifery, Students Research Committee, Midwifery Department, Faculty of Nursing and Midwifery, Tabriz University of Medical sciences, Tabriz, Iran; 2 Cabrini Research, Cabrini Health, Malvern, VIC 3144 Australia; 3 School of Public Health and Preventative Medicine, Faculty of Medicine, Nursing and Health Sciences, Monash University, VIC, 3800 Australia; 4 Road Traffic Injury Research Center, Tabriz University of Medical Sciences, Tabriz, Iran; 5 Department of Psychiatry, Faculty of Medicine, Ardabil University of Medical Science, Ardabil, Iran; 6 Students Research Committee, Midwifery Department, Faculty of Nursing and Midwifery, Tabriz University of Medical sciences, Tabriz, Iran; 7 Department of Midwifery, Faculty of Nursing and Midwifery, Tabriz University of Medical Sciences, Tabriz, Iran; 8 Reproductive Health Research Center, Midwifery Department, Faculty of Nursing and Midwifery, Urmia University of Medical Sciences, Urmia, Iran; 9 Social Determinants of Health Research Center, Department of Midwifery, Faculty of Nursing and Midwifery, Tabriz University of Medical Sciences, Tabriz, Iran

**Keywords:** Validation study, World fertility surveys, Marital fertility, Fertility preferences

## Abstract

**Background:**

One of the most important population challenges during the last three decades has been the significant decrease in the fertility rate worldwide. Since the validity and reliability of the Male and Female Fertility Knowledge Inventories (MFKI and FFKI) have not been determined in Iran, we conducted this study to assess psychometric characteristics of the MFKI and FFKI in couples in Tabriz and Urmia, Iran.

**Methods:**

This was a cross-sectional study, as the first part (quantitative phase) of a sequential explanatory mixed-method study. The current study was done on 1200 participants (600 women with their husbands) living in the northwestern region of Iran, between January 2023 and September 2023. The psychometric properties of the Persian version of the tool (MFKI and FFKI) were performed in 5 stages, including translation process, content validity, face validity, construct validity and reliability assesment.

**Results:**

In this study, the CVI, CVR, and impact scores of the MFKI tool were equal to 0.90, 0.88, 3.26 and CVI, CVR, and Impact scores of the FFKI tool were respectively equal to 0.95, 0.91 and 3.59, that it indicated a satisfactory level of content and face validity. Then, to check the construct validity, the results of the exploratory factor analysis of the MFKI tool on 13 items led to the identification of 3 factors, including Environment and reproductive health (ERH), Lifestyle factors (LSF) and Sperm quality (SQ), which explained 66% of the cumulative variance. The results of the exploratory factor analysis of the FFKI tool on 15 items led to the identification of 4 factors, including Reproductive health (RH), Lifestyle factors (LSF), Chance of conception (CHC) and Ovarian reserve and preservation (ORP), which explained 68% of the cumulative variance.

**Conclusions:**

The findings of this study indicated that the Persian version of MFKI and FFKI has acceptable psychometric properties to measure the awareness of Iranian couples regarding fertility, which can be used as a screening tool for fertility knowledge by health care professionals and also as a reliable tool in research.

## Abstract (plain language summary)

The right to sexual and reproductive health (SRH) is considered one of the basic rights of couples all over the world. Increasing childbearing age is a global social issue. In spite of the policies of having children in the previous decade, fertility reduction and involuntary childlessness are still the foremost global health problems. The fertility rate in Iran has reduced significantly in the last thirty years. This reduction can be attributed not only to economic difficulties but also to the postponement of marriage and having children, as well as infertility issues, which, by increasing the awareness of couples about fertility, seems to be able to overcome this problem to some extent. Consequently, we decided to conduct this study with the aim of determining the psychometric properties of the Male and Female Fertility Knowledge Inventories (MFKI and FFKI) in Iranian couples. The results of the current study revealed that this questionnaire, having acceptable psychometric properties to evaluate the state of awareness of Iranian couples regarding fertility, can be used as a valid and reliable tool in Iranian couples. It appears that it is essential for health care providers to play an active role in advising couples about the appropriate age of fertility, the problems of delay in having children, overcoming the value of having few children in society. Accordingly, health policymakers should recommend the use of valid screening tools to identify the knowledge of women and men about fertility in health centers.

## Background

Fertility and reproduction, a physiological process in a couple's life, is considered one of the most important goals of every marital union and the basis of human survival [1]. Deciding to have children is one of the major events in a couple's life, affecting many aspects of life, such as health, economic status, and family well-being [2]. Generally, demographic changes are influenced by fertility, mortality, and migration. From the demographic perspective, fertility is known as the most important phenomenon that determines population fluctuations (age and gender composition of the population), and related studies are more important than other demographic phenomena (death and migration) [3].

One of the most significant demographic challenges in the last three decades has been the dramatic decline in fertility rates. The total fertility rate in the world has reached from 6 children per woman in 1960 to 2.5 births in 2013 and 2.4 births per woman in 2020, which indicates a global decrease in fertility [4, 5]. Iran is not an exception to this rule. Since 2006, Iran's total fertility rate has been decreasing alarmingly. In contrast, between 2006 and 2011, the annual population growth rate increased from 2.6 to 29.1. As reported in the population census of Iran in 2011, the total fertility rate has reached its peak at 7.1 children per woman. Subsequently, the total fertility rate of the population of Iran gradually decreased and reached 1.69 children per woman in 2014, then 1.61 children per woman in 2018 and 2.2 children per woman in 2020 [6].

Based on the World Bank's estimate, the growth rate of Iran's population during 2015-2019 will be equal to 1.32% and reach 1.13% and 1% during 2020-2024 and 2025, respectively [7]. Fertility postponement can lead to serious problems, especially in the economic field, including reducing the labor force, which can negatively affect investment. Furthermore, society will face an aging population shortly, imposing heavy costs on society [3]. From the social aspect, there will be a generation gap and a change in the family structure. Consequently, reducing the desire to have children has also been added to the list of country issues that need solutions [8].

A widespread range of social, economic, personal, religious, and cultural factors, including access to effective methods of contraception and safe abortion, social changes such as increasing the level of women's education and participation in the labor market, postponing marriage, value changes and structural factors such as rising housing costs, economic uncertainty, etc. existence of family support policies and job security also affect the timing of having children [9, 10]. Some potentially adjustable factors, including parental obesity, smoking, and the advanced age of the couple, are associated with reduced fertility, neonatal complications, and subsequently increased use of assisted reproductive technologies (ART). Consequently, the consequences of reducing the fertility rate for society, including the reduction of the labor force, the aging population in the future, personal and psychological costs related to infertility due to increasing age, and the financial costs of assisted reproductive methods for couples and the health care system are significant [11].

Factors involved in the fertility rate include external and internal factors. External factors include educational policies, facilities, religious beliefs, economic conditions, marriage age, family members' roles, and health care providers' roles. In recent years, not only external factors but also the role of internal factors in shaping behavior, including the role of couples' desires and awareness regarding fertility and the disadvantages of delaying it, have been extremely considered [12, 13].

Many studies reveal that awareness of fertility is an important factor in the tendency and formation of fertility behavior. Fertility awareness is defined as a person's level of understanding about couples' fertility decline with age, infertility risk factors, and misconceptions about fertility [14]. Based on the results of studies, the level of awareness and fertility knowledge of couples in the general population is weak. Evidence indicates that couples are unaware of the biological aspects of conception. They often overestimate the chances of getting pregnant when they ovulate and mistakenly equate being healthy with being fertile. They do not have a proper understanding of the sharp decline in fertility of women with increasing age and the specific risk factors involved in the decline of fertility, including sexually transmitted diseases (STDs), smoking, and alcohol consumption [15].

Consequently, understanding the internal factors involved in the delay in childbearing, including the lack of knowledge and awareness of couples, is largely the reason for this decline in childbearing. The multi-factor decision-making process of delay in childbearing related to the job, education level, financial security, and health is a reason for not recognizing the contribution of knowledge and awareness of couples in childbearing behaviors [16].

Consequently, health counseling provided by gynecologists and midwives is necessary to improve the understanding of couples about fertility behavior, the harms of delayed pregnancy, and the timing of pregnancy. Before designing any intervention to increase the fertility rate, it seems necessary to measure the knowledge of couples in society with valid and reliable tools. In spite of the multitude of scales, no scale measures the special fertility factors of men and women separately. The extensively used tool available to measure the knowledge of couples is the Cardiff Fertility Knowledge Scale (CFKS) with 13 questions, which is considered the same for men and women and is not separated [17]. Since some specific factors affect one gender more than the other (for example, the greater effects of age on female fertility), it is necessary to have a particular tool for each gender. The Fertility Knowledge Inventory tool was designed in 2018 by Olekalns et al. Olekalns has overcome the problem of the previous tools by creating two separate tools for measuring fertility knowledge in women and men (Female Fertility Knowledge Inventory (FFKI) and Male Fertility Knowledge Inventory (MFKI). The FFKI tool has 15 items and four factors, including Reproductive health (RH), Lifestyle factors (LSF), Chance of conception (CHC) and Ovarian reserve and preservation (ORP). Similarly, the MFKI tool has 14 items and 3 factors, including Environment and reproductive health (ERH), Lifestyle factors (LSF) and Sperm quality (SQ) [14].

These two instruments have not been psychometrically evaluated in Iran. Likewise, because of the demographic changes that have occurred in the whole world, particularly in Iran and the unprecedented decrease in childbearing during the last three decades, the lack of attention and addressing the problem of continuous reduction of fertility and the transition from natural fertility to controlled fertility is gradually changing the age structure of the population from young to old. Consequently, we decided to conduct this study with the aim of determining the psychometric properties of the MFKI and FFKI in Iranian couples (women and their husbands).

## Methods

### Study participants and setting

The present study, as the first part (quantitative phase) of a sequential explanatory mixed-method study was conducted from January 2023 to September 2023. The permission was received from the Ethics Committee of Tabriz University of Medical Sciences (Ethical approval code: IR.TBZMED.REC.1401.211). To determine the psychometric characteristics of the Persian version of the MFKI and FFKI, two steps were used to validate and determine the psychometric properties of MFKI and FFKI. In the first stage, the translation of the tool and the pilot were done for content validity, face validity, and reliability evaluation. The second step was to evaluate the psychometric properties with a significant sample size to check the construct validity.

### The psychometric evaluation of the scale

#### Translation procedure

After acquiring permission from the main designers of the tool (Olekalns et al.) [14], the translation process was done based on the World Health Organization (WHO) guideline [18] and using the dual panel approach, and Guidelines for the Process of Cross-Cultural Adaptation of Self-Report Measures. In the first approach, the translation process was done in three stages [19]. The first panel consisted of a group of 10 expert panel include: Three expert in Midwifery, two expert in reproductive health, one clinical psychologist, one expert in Medical Surgical Nursing, two expert in Psychiatric Nursing and one expert in Community Health Nursing. The second panel (layman panel) consisted of 10 eligible women and their husbands. In the third stage (the target group panel), 1200 participants (600 women and their husbands) completed the questionnaire anonymously. The second approach occurred during five stages, including, Stage I: Initial Translation, Stage II: Synthesis of The Translations, Stage III: Back Translation, Stage IV: Expert Committee, Stage VI: Pretesting [20].

In the first step, the original version of the tool in the Farsi language was translated independently by two Farsi-speaking people who are fluent in English and experts in the field of tool preparation and childbearing. Then, the two translators compared their translated versions with each other, and after resolving the conflicts, they presented a final version. In the second step, to completely ensure the conformity of the Persian translation and the accuracy of the sentences of the Persian text, the final version created in the Persian language in the previous step was translated back into English by two people who had not seen the original version and were not involved in the translation process of the original version. Then, the two English backward and original versions were compared to check the transfer of the same concepts. Lastly, the translated questionnaire was given to 10 Iranian couples (10 qualified women and their husbands) for a preliminary evaluation of the comprehensibility of the questions and the simplicity of the concepts. Finally, based on the opinions of women and their husbands, changes were made in the Persian version regarding the fourth criterion, including Semantic Equivalence, Idiomatic Equivalence, Experiential Equivalence, and Conceptual Equivalence [21].

#### Content validity

To check the content validity, a qualitative method was used based on the evaluation of the expert committee, and a quantitative approach was used based on the calculation of the content validity ratio (CVR) and content validity index (CVI). In the qualitative method, the opinions of 10 experts were collected regarding the content, Persian language grammar, appropriateness with social-cultural characteristics, and adding new items, and then, based on their feedback, corrections were made, and the questionnaire was edited [22].

For quantitative evaluation, CVI was evaluated based on the content validity index of Waltz and Basel [23]. In this regard, questions were asked about each item's three criteria of relevance, clarity, and simplicity based on a four-point Likert scale, in which experts determined the degree of relevance, clarity, and simplicity. For example, the response options for simplicity were “1= not simple”, “2= somewhat simple, “3= simple” and “4= quite simple”. To this end, CVI scores were the sum of the 3 and 4 (highest possible) marks given by the experts to each item divided by the number of experts. Acceptance of items based on CVI score was higher than 0.79 [24]. Then, in the next step, to determine the CVR, questions were asked about the necessity of each item based on a three-point Likert scale (necessary, useful but unnecessary, unnecessary), and it was calculated using the formula $CVR=\frac{Ne-N/2}{N/2}$. CVR > 0.78 confirmed the necessity of items [25].

#### Face validity

For face validity, quantitative and qualitative approaches were used. The quantitative approach was evaluated by calculating the impact score, and the qualitative approach was based on the opinions of the expert committee and target groups' views [26]. Questionnaire items in the face validity form include the first part (qualitative evaluation), checking in terms of difficulty levels, irrelevance, and ambiguity. The second part (quantitative evaluation) was included in calculating the impact score, checking the importance of the items based on a 5-point Likert scale (completely important, important, moderately important, slightly important, and not important). Then, the convenience sampling questionnaire was given to 10 eligible women and their husbands. Lastly, the score of each item was calculated using the following formula: Impact Score = Frequency (%) × Importance. Finally, the items with an impact score of more than 1.5 were accepted [27].

#### Construct validity (exploratory and confirmatory factor analysis)

For construct validity, a cross-sectional study was carried out on 1200 participants (600 women with their husbands) living in the northwestern region of Iran (Tabriz and Urmia health centers), via cluster random sampling. The samples size to perform construct validity in factor analysis is 5 to 10 for each item. Based on the rule of thumb, the sample size for exploratory factor analysis is classified as 50= very poor, 100= poor, 200= fair, 300= good, 500= very good and 1000= excellent [28]. The sample size in this study is equal to 1200 participant (600 women and 600 of their husbands) who were considered for exploratory and confirmatory factor analysis. To determine the sample size in this study, the sample size was calculated based on the results of Olekalns et al.'s study [14] about men's knowledge of fertility and considering Mean=8.86, SD=3.05, d=0.05 and Alpha=0.05 equal to 182 participant and about women's knowledge about fertility and with Considering Mean=10.35, SD=3.19, d=0.05 and Alpha=0.05, it was calculated equal to 146 participant. Due to the larger sample size calculated based on the knowledge of men and cluster sampling (Design effect = 1.5) and considering the 10% possible dropout, the final sample size was estimated to be 300 participant. Three hundred couples (300 women and 300 husbands) were examined in each city (Tabriz and Urmia).

In the present study, to do the sampling process, sampling was done in two centers of East Azerbaijan (Tabriz) and West Azerbaijan (Urmia). The cluster random sampling method was used for sampling in each province center. First, a quarter of the centers were randomly selected using the www.random.org website, then the researcher went to the chosen centers and extracted the list of women along with their telephone numbers from the SIB system (integrated health system), the number of women selected from each center was proportionally calculated concerning the sample size. The researcher made a phone call with the chosen women and, during the same phone call, checked the women and their husbands in terms of inclusion and exclusion criteria, and if they were eligible to enter the research, the researcher offered them information about the research, how to conduct it and the confidentiality of the information, and offered them to participate in the study. If the couples agreed to participate in the study, they were asked to be present at the health center with their husbands at a certain time. In the face-to-face visit, informed consent to participate in the research was obtained from the participants. The aims and methods of the study were fully explained to all eligible couples, and if they were willing to participate in the study, written informed consent was obtained from them. The questionnaires of socio-demographic characteristics, MFKI and FFKI were completed anonymously by the couples.

The inclusion criteria consisted of married women and men living in the northwestern region of Iran, being in reproductive age (women aged 18-49 and men aged 18-59), not having a history of primary infertility, women and men who have been married for more than a year and do not have children, and women and men who have passed more than three years since their last child. Couples having more than one child, widowed or divorced women and men, mental illness in each of the couples, taking antidepressants, including tricyclic antidepressants (TCAs), selective serotonin reuptake inhibitors (SSRIs), monoamine oxidase inhibitors (MAOIs), and noradrenaline (NA), stressful events such as divorce, death of first-degree family members, and diagnosis of an incurable disease for one of the family members during the last three months, and the presence of a specific or chronic illness such as cancer or heart and kidney diseases in each of the couples were excluded from the present study.

#### Measures

The two questionnaires used in this study included socio-demographic questionnaire, MFKI or FFKI.

##### Socio-demographic questionnaire

The socio-demographic questionnaire included information such as age, husband's age, marriage age, marriage duration, gravidity, number of abortions, type of childbirth, woman's education, occupation, monthly income adequacy, marital satisfaction, smoking status, exercise, contraception, assisted reproductive methods, husband's occupation, and husband's education.

##### Male and Female Fertility Knowledge Inventories (MFKI and FFKI)

The Male and Female Fertility Knowledge Inventories (MFKI and FFKI) tools were designed separately by Olekalns et al. (2018) in Australia to measure men's and women's fertility knowledge. The MFKI tool is special for measuring men's knowledge of fertility, which has 14 items and three factors, including Environment and reproductive health (ERH) (5 items), Lifestyle factors (LSF) (4 items) and Sperm quality (SQ) (5 items). The FFKI tool is also special for measuring women's knowledge of fertility, which has 15 items and four factors, including Reproductive health (RH) (3 items), Lifestyle factors (LSF) (4 items), Chance of conception (CHC) (3 items) and Ovarian reserve and preservation (ORP) (5 items). In men and women, the tools are completed in the form of a three-point Likert scale (True, false, and I don't know). The true answer gets a score of 1, false, and I don't know choices are given zero scores. Higher scores indicate a higher knowledge of women and men regarding fertility. The validity and reliability of these tools have been proven in the Australian male and female population, respectively, with Cronbach's alpha coefficient of 0.78 and 0.77 [14].

Construct validity was examined using exploratory factor analysis (EFA), with Kaiser-Meyer Olkin (KMO) and Bartlett's test of Sphericity criteria. Then, the principal component analysis method with Varimax rotation (direct oblimin) was used to extract the factors. Likewise, the factor load value was considered above 0.3 [29]. In confirmatory factor analysis (CFA) using JASP 1.18 software, a series of indicators such as Root Mean Square Error of Approximation < 0.08, Standardized Root Mean Square Error of Approximation (SRMSEA) < 0.08, Normed Chi2 (x2 / df) < 5, comparative fit indices including comparative fit index (CFI > 0.90) and Tucker-Lewis Index (TLI) > 0.90, were used to check the suitability of the model [30, 31].

#### Reliability

Finally, to determine the reliability of the questionnaire, internal consistency and test-retest reliability were used [32, 33]. Internal consistency was checked by determining Cronbach's alpha and McDonald's omega coefficients for each factor and the whole tool. To assess the stability of retesting, the questionnaire was completed by 40 people (20 women and 20 men separately) via random sampling in two stages with a time interval of two weeks to determine the intra-class correlation coefficient (ICC). ICC between 0.6-0.8 was considered good, and above 0.8 as excellent. Similarly, Cronbach's alpha and McDonald's omega coefficients above 0.7 were satisfactory [34].

#### Ethical considerations

The Ethics Committee of Tabriz University of Medical Sciences approved the current study (Ethical approval code: IR.TBZMED.REC.1401.211). After getting the required permission from the main designers of the tool (Olekalns et al.), written informed consent was obtained from all participants. All couples were given full explanations about the process and objectives of the study, confidentiality of information, preservation of privacy, and freedom to withdraw at each stage of the study. This study was done based on the World Medical Association of Helsinki declaration.

### Statistical analysis

In the current study, SPSS Statistics 14 (IBM Corp, Armonk, NY, USA), STATA 14 (Statcorp, college station, Texas, USA) and R software 4.2 (Psych package) were used for data analysis. To describe socio-demographic characteristics, data were presented as mean (SD) and frequencies (%) for quantitative and qualitative variables, respectively. Content and face validity (CVR and CVI, Impact score), EFA, and CFA were also determined for construct validity. The reliability of the tool was also calculated through Cronbach's alpha coefficient, McDonald's omega and ICC.

## Results

### General characteristics of participants

A total of 1200 (600 women and 600 husbands) participants entered the present study. The mean (SD: standard deviation) age of women and their husbands was 30.9 (6.1) and 34.8 (5.6) years, respectively. Over half of the women (63.0%) stated only one pregnancy history. Likewise, about half of the women (50.8%) had a history of cesarean delivery. About two-thirds of women (67.5%) were housewifes. More than two-thirds of women (72.3%) used contraceptive methods. Less than half of the women (45.6%) had a diploma, while about half of the husbands (51.6%) had a university education. Most women and their husbands (89.9% and 85%, respectively) were completely satisfied with their married life. Other demographic characteristics of the participants are summarized in Table 1 (Table 1).

**Table 1.** Baseline characteristics of participants for factor analysis of Male and Female Fertility Knowledge Inventories (*n* = 1200 (male= 600, female=600))

**Characteristics**	**Mean**	**SD**^a^
**Age (Years)**	30.9	6.1
**Husband’s age (Years)**	34.8	5.6
**Marriage age (Years)**	24.6	6.7
**Marriage duration (Years)**	6.3	4.4
	**Number**	**Percent**
**Gravidity**		
**0**	137	22.5
1	384	63.0
2	78	12.8
≥3	11	1.8
**Abortion history**		
0	494	81.0
1 and more	116	19.0
**Delivery type**		
No	129	21.1
NVD with Epi	25	4.1
NVD without Epi	58	9.5
Instrumental delivery	88	14.4
C/S	310	50.8
**Education**		
Illiterate or elementary	16	2.6
Secondary or high school	84	13.8
Diploma	278	45.6
University	231	37.9
**Job**		
Housewife	412	67.5
Employee	198	32.5
**Income**		
Inadequate	221	36.2
Relatively adequate	266	43.6
Completely adequate	123	20.2
**Marital satisfaction**		
Not at all	62	10.2
Relatively	273	44.8
Completely	275	45.1
**Women's smoking**		
Yes	92	15.1
No	518	84.9
**Exercise**		
No	196	32.1
Low	219	35.9
Modereate/ Prefessional	195	31.9
**Contraception**		
Yes	441	72.3
No	169	27.7
**ART**		
Yes	70	11.5
No	540	88.5
**Husband’s Job**		
Labor	130	21.3
Employee	174	28.5
Self-employed	286	46.9
Professionalist/ Manager	20	3.3
**Husband’s education**		
Illiterate or elementary	37	6.1
Secondary or high school	132	21.6
Diploma	126	20.7
University	315	51.6

^a^Standard deviation; Data presented as *n* (%)

The mean (SD) of the entire MFKI scale in this study was equal to 7.9 (3.4), and for the three extracted factors, including ERH, LSF, and SQ, it was equal to 2.6 (1.4), 2.6 (1.3) and 2.7 (1.4) respectively. Similarly, the mean (SD) of the entire FFKI scale in this study is equal to 8.6 (3.5), and for the four extracted factors, including RH, LSF, CHC, and ORP, it is equal to 2.1 (1.1), 2.3 (1.2), 1.2 (1.3) and 2.9 (1.3) respectively.

### Content and face validity

In the content validity check, all the items obtained the acceptable value of CVI and CVR, which was equal to 0.90 and 0.80 in the MFKI tool (Table 2). It was also equal to 0.95 and 0.91 in the FFKI tool (Table 3). In addition, the face validity examination revealed that the items have acceptable face validity and received a minimum score of 1.5 (Tables 2 & 3).

**Table 2:** The results for the content and face validity of the MFKI (*n*=10)

**Item**	**CVI**				**CVR**	**Impact score**
	**Simpility**	**Clarity**	**Relevance**	**Total score**		
MFKI1	0.90	0.90	0.90	0.90	0.80	1.90
MFKI2	0.80	0.80	1.00	0.86	0.80	2.72
MFKI3	1.00	1.00	1.00	1.00	1.00	4.00
MFKI4	0.80	0.70	0.90	0.80	0.60	3.40
MFKI5	0.70	0.70	0.80	0.73	0.60	2.20
MFKI6	1.00	1.00	1.00	1.00	1.00	4.00
MFKI7	1.00	1.00	1.00	1.00	1.00	3.60
MFKI8	1.00	1.00	1.00	1.00	1.00	2.90
MFKI9	0.90	0.90	1.00	0.93	1.00	4.00
MFKI10	0.80	0.70	0.90	0.80	0.90	3.90
MFKI11	1.00	1.00	1.00	1.00	1.00	4.00
MFKI12	0.90	1.00	1.00	0.96	0.80	2.90
MFKI13	0.90	0.90	1.00	0.93	1.00	3.50
MFKI14	0.70	0.70	0.70	0.70	0.80	2.60
Total	0.88	0.87	0.94	0.90	0.88	3.26

*CVI* Content Validity Index, *CVR* Content Validity Ratio, *MFKI* Male Fertility Knowledge Inventories

**Table 3:** The results for the content and face validity of the FFKI (*n*=10)

**Item**	**CVI**				**CVR**	**Impact score**
	**Simpility**	**Clarity**	**Relevance**	**Total score**		
FFKI1	0.90	0.90	1.00	0.93	1.00	3.70
FFKI2	0.90	0.90	1.00	0.93	0.80	1.60
FFKI3	0.90	0.90	1.00	0.93	1.00	4.00
FFKI4	1.00	1.00	1.00	1.00	1.00	4.00
FFKI5	1.00	1.00	1.00	1.00	1.00	3.00
FFKI6	1.00	1.00	1.00	1.00	1.00	4.00
FFKI7	0.90	0.90	1.00	0.93	1.00	4.00
FFKI8	0.80	0.80	0.90	0.83	0.80	2.70
FFKI9	0.90	0.90	0.90	0.90	0.80	4.00
FFKI10	0.90	0.90	0.90	0.90	0.80	2.80
FFKI11	1.00	1.00	1.00	1.00	1.00	4.00
FFKI12	0.90	0.80	1.00	0.90	0.80	4.00
FFKI13	0.90	0.90	1.00	0.93	0.80	4.00
FFKI14	1.00	1.00	1.00	1.00	0.80	4.00
FFKI15	1.00	1.00	1.00	1.00	1.00	4.00
Total	0.93	0.93	0.98	0.95	0.91	3.59

*CVI* Content Validity Index, *CVR* Content Validity Ratio, *FFKI* Female Fertility Knowledge Inventories

### Construct validity

#### Exploratory factor analysis

**Fig. 1:**
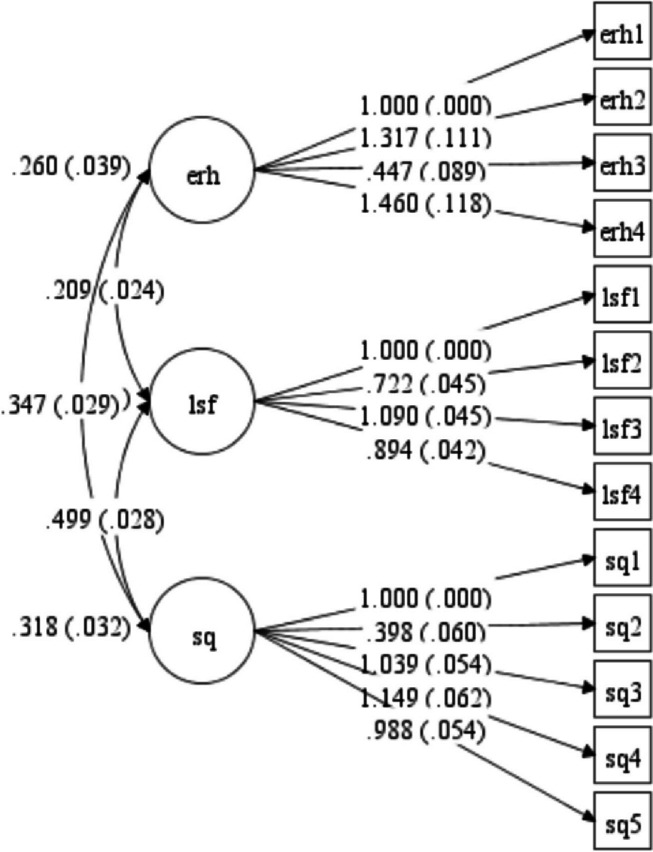
Factor structure model of the MFKI based on CFA. (All factor loadings are significant at *p* < 0.001). MFKI: Male Fertility Knowledge Inventories, ERH: Environment and reproductive health, LSF: Lifestyle factors, SQ: Sperm quality

In the investigation of construct validity, during the process of exploratory factor analysis, in the MFKI tool, a 3-factor structure with a total variance of 66% was obtained (Table 4). The first factor was ERH, which consisted of 5 questions that accounted for 23% of the total variance. During the process of exploratory factor analysis, question 3 was removed due to factor loading less than 0.3. Finally, the number of questions was reduced from 5 to 4 questions. The second factor is LSF, which has four questions, explaining 22% of the total variance. The third factor is SQ, which has five questions explaining 21% of the total variance (Fig. 1).

**Table 4:** Result of Facture analysis of the MFKI based on EFA (*n*=600)

**Scale item**	**Factors**		
	**1**	**2**	**3**
**Factor 1: Environment and reproductive health (ERH)**			
1. An occlusion (blockage) in the male reproductive system can affect a man’s fertility	0.537		
2. Men do not experience a natural decline in their fertility	0.378		
3. If a man already has one biological child, he will not have trouble conceiving again	0.646		
4. Use of anabolic steroids once a week can negatively affect a man’s fertility (steroids contain Testosterone and are performance enhancing drugs used to increase muscular strength and body weight)	0.848		
**Factor 2: Lifestyle factors (LSF)**			
5. A man’s weight/BMI (Body Mass Index) can affect his fertility		0.858	
6. A man's diet does not affect his fertility		0.686	
7. Chronic consumption of alcohol can affect sperm quality		0.913	
8. Smoking cigarettes can affect a man’s fertility		0.668	
**Factor 3: Sperm quality (SQ)**			
9. Men continue to produce and mature new sperm every 72 days			0.805
10. Intense, sustained exercise can improve a man’s sperm quality (i.e., 4-5 times a week for 2 hours)			0.328
11. Men who have had mumps before puberty may experience fertility problems if left untreated			0.589
12. Some lubricants negatively affect sperm			0.697
13. Chromosomal changes can affect the production and transportation of sperm			0.616
**% of variance observed**	0.23	0.22	0.21
**Total score**	0.66

**Fig. 2:**
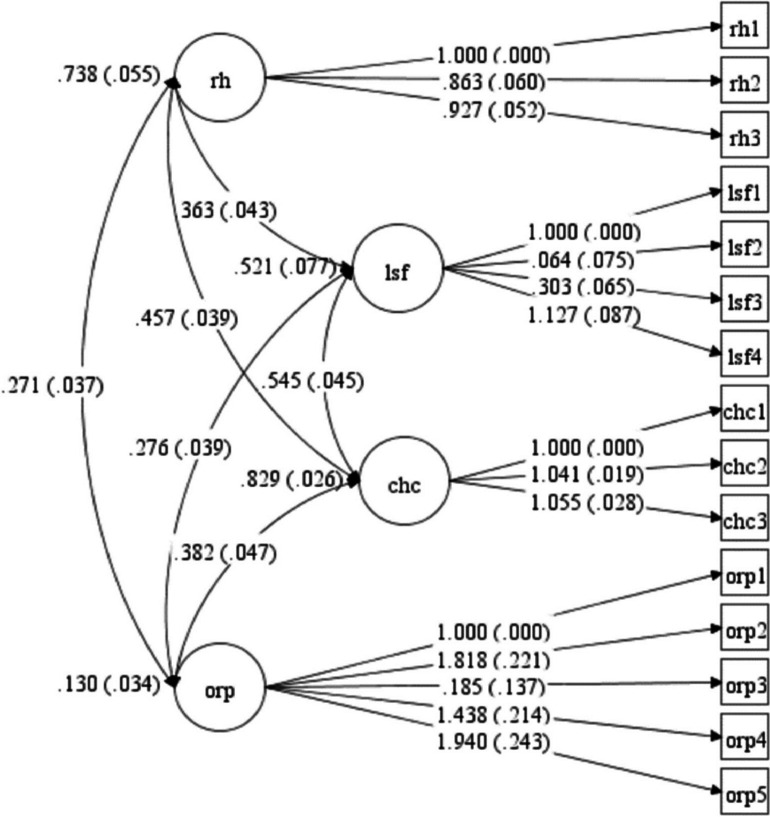
Factor structure model of the FFKI based on CFA. (All factor loadings are significant at *p* < 0.001). FFKI: Female Fertility Knowledge Inventories, ERH: Environment and reproductive health, LSF: Lifestyle factors, SQ: Sperm quality, RH: Reproductive health, CHC: Chance of conception, ORP: Ovarian reserve and preservation

In the FFKI tool, a 4-factor structure with a total variance of 68% was obtained (Table 5). The first factor was RH, which included three questions that accounted for 24% of the total variance. The second factor is LSF, which has four questions, explaining 21% of the total variance. The third factor, CHC, has three questions that explain 11% of the total variance. Finally, the fourth factor was ORP, which consisted of 5 questions that accounted for 10% of the total variance (Fig. 2).

**Table 5:** Result of Facture analysis of the FFKI based on EFA (*n*=600)

**Scale item**	**Factors**			
	**1**	**2**	**3**	**4**
**Factor 1: Reproductive health (RH)**				
1. Sexually transmitted infections, including chlamydia, gonorrhoea and HPV (Human Papillomavirus), can affect a woman’s fertility	0.795			
2. A history of endometriosis can affect a woman’s fertility	0.959			
3. A woman’s weight/BMI (Body Mass Index) can affect her fertility	0.567			
**Factor 2: Lifestyle factors (LSF)**				
4. A woman's diet does not affect her fertility		0.424		
5. Toxins in the environment (i.e., chemicals, pesticides, heavy metals) can affect a woman’s fertility		0.569		
6. Smoking cigarettes can affect a woman’s fertility		0.999		
7. Moderate, sustained exercise can improve a woman's fertility (i.e., up to 4 hours of brisk walking a week)		0.306		
**Factor 3: Chance of conception (CHC)**				
8. The risk of miscarriage for fit and healthy women is the same, whether they are in their 30s or their 40s			0.951	
9. A woman in her 40s is equally as likely to become pregnant through IVF as a woman in her 30s			0.953	
10. More than half of women and their partners conceive on the first round of IVF			0.897	
**Factor 4: Ovarian reserve and preservation (ORP)**				
11. Women continue to produce new eggs until they reach menopause				0.534
12. The primary role of a fertility specialist is to provide IVF (In Vitro Fertilisation) to a woman				0.584
13. A woman who has a regular menstrual cycle is fertile				0.361
14. Taking vitamin supplements can increase a woman’s ovarian reserve (the number of eggs available to her, and the number of fertile years she has remaining)				0.343
15. Freezing her eggs guarantees a woman will be able to become pregnant in the future				0.891
**% of variance observed**	0.24	0.21	0.11	0.10
**Total score**	0.68

#### Confirmatory factor analysis

In the confirmatory factor analysis, three factors obtained from the MFKI tool were confirmed in exploratory factor analysis by CFA (RMSEA = 0.08, SRMR = 0.09, CFI = 0.90, TLI = 0.90, x_2_/df (normed chi-square) = 2.40) (Table 6). Also, the obtained four factors of the FFKI tool were confirmed using CFA (RMSEA = 0.08, SRMR = 0.07, CFI = 0.97, TLI = 0.91, x_2_/df (normed chi-square) = 1.68) (Table 7). Based on the results, these models achieved a satisfactory level of fit, based on which the factorial structure can be confirmed.

**Table 6:** The model fit indicators of the MFKI (*n*=600)

**Goodness of fit indices**	**CFA**	**Acceptable value**
**χ2**	184.87	
**Df**	74	
$^{{{\varvec{x}}}^{2}}\!\left/ \!_{{\varvec{d}}{\varvec{f}}}\right.$	2.40	<5
***P*-value**	<0.001	0.05>
**CFI**	0.90	>0.90
**TLI**	0.90	>0.90
**SRMR**	0.09	<0.10
**RMSEA (90% CI)**	0.08	<0.08

*χ2* Chi-square, *df* Degrees of freedom; χ2/df Normed chi-square, *CFI* Comparative Fit Index, *TLI* Tucker–Lewis index, *SRMR* Standardized root mean squared residual, *RMSEA* Root mean square error of approximation

**Table 7:** The model fit indicators of the FFKI (*n*=600)

**Goodness of fit indices**	**CFA**	**Acceptable value**
**χ2**	150.90	
**Df**	84	
$^{{{\varvec{x}}}^{2}}\!\left/ \!_{{\varvec{d}}{\varvec{f}}}\right.$	1.68	<5
**P-value**	<0.001	0.05>
**CFI**	0.97	>0.90
**TLI**	0.91	>0.90
**SRMR**	0.07	<0.10
**RMSEA (90% CI)**	0.80	<0.08

*χ2* Chi-square, *df* Degrees of freedom, *χ2/df* Normed chi-square; *CFI* Comparative Fit Index, *TLI* Tucker–Lewis index; *SRMR* Standardized root mean squared residual, *RMSEA* Root mean square error of approximation

#### Reliability

To determine the reliability of the tool, Cronbach's alpha coefficient and McDonald's omega calculated for the entire MFKI tool were 0.78 and 0.80, respectively. Similarly, for the FFKI tool, it was equal to 0.78 and 0.79, respectively, indicating both tools' good internal consistency. Also, in the test-retest method, ICC (95% CI) for MFKI and FFKI tools, respectively, is equal to 0.91 (0.77 to 0.97), 0.98 (0.94 to 0.99) respectively (Table 8).

**Table 8:** Stability Coefficients and Interclass Correlation Coefficient of the MFKI and FFKI

**Factors**	**Cronbach’s α coefficient**	**McDonald's omega**	**ICC (95% CI)**
ERH	0.76	0.75	0.78 (0.44 to 0.91)
LSF	0.86	0.84	0.90 (0.74 to 0.96)
SQ	0.92	0.92	0.97 (0.93 to 0.99)
MFKI (Total)	0.78	077	0.91 (0.77 to 0.97)
RH	0.95	0.95	0.91 (0.77 to 0.96)
LSF	0.86	0.84	0.93 (0.83 to 0.97)
CHC	0.92	0.92	0.93 (0.82 to 0.97)
ORP	0.87	0.89	0.99 (0.96 to 0.99)
FFKI (Total)	0.78	077	0.98 (0.94 to 0.99)

*ICC* intra class correlation coefficient, *CI* Confidence interval, *MFKI* Male Fertility Knowledge Inventories, *ERH* Environment and reproductive health, *LSF* Lifestyle factors, *SQ* Sperm quality, *FFKI* Female Fertility Knowledge Inventories, *ERH* Environment and reproductive health, LSF: Lifestyle factors, SQ: Sperm quality, *RH* Reproductive health, *CHC* Chance of conception, *ORP* Ovarian reserve and preservation

## Discussion

The results of the current study with the aim of psychometric evaluation of MFKI and FFKI in Iranian couples, revealed that this questionnaire, having acceptable psychometric properties to evaluate the state of knowledge of Iranian couples regarding fertility, can be used as a valid and reliable tool in Iranian couples. The right to sexual and reproductive health (SRH) is considered one of the basic rights of couples all over the world. The International Conference on Population and Development (ICPD) describes reproductive rights based on “the fundamental right of all couples to make free and responsible decisions about the number, spacing, and time of having children and to have the information and tools to do so” (http://www.euro.who.int/en/health-topics/Life-stages/sexual-and-reproductive-health/sexual-and-reproductive-healthWHOSarhIWHOcFpAf).

The MFKI and FFKI scales were the first designed specifically and separately for men and women, respectively, covering the areas of Environment and reproductive health (ERH), Lifestyle factors (LSF) and Sperm quality (SQ) in men and Reproductive health (RH), Lifestyle factors (LSF), Chance of conception (CHC) and Ovarian reserve and preservation (ORP) in women, to screen couples' knowledge of fertility. In the current study, the three extracted factors of the exploratory factor analysis process for the 13 items of the MFKI tool, including ERH, LSF, and SQ, which explained 66% of the variance, which was 46.23% in the original scale [14]. Besides, four factors were extracted for 15 items of the FFKI tool, including RH, LSF, CHC, and ORP, which explained 68% of the variance, which was equal to 53.74% in the original scale [14]. To confirm the validity of the tool, the value of KMO and the significance of Bartlett's test also confirmed the adequacy of the model.

The factors extracted from the FFKI questionnaire agree with the results of some studies in this field. The first factor extracted in this study was reproductive health (RH). Reproductive health is the main science of human life and is vital for the sustainable development of human society. Based on the WHO report, issues related to sexual health and fertility constitute more than a third of the global burden of diseases in women. In women, 36% of healthy life is lost due to reproductive health problems such as maternal mortality, pregnancy complications, and sexually transmitted diseases (STDs) [35]. The two main causes of poor reproductive health in women are lack of knowledge and lack of access to health services [36]. In this regard, the results of Chen et al.'s (2020) study on the state of reproductive health and factors related to the level of awareness among women of reproductive age in China disclosed that the scores of awareness of reproductive health were low in more than half of the women. Similarly, a statistically significant relationship was observed between factors such as education level, history of gynecological disease, and knowledge attained through medical staff or the Internet with the level of women's awareness of their reproductive health [37].

The second factor extracted in this study was Lifestyle Factors (LSF). Lifestyle factors refer to changeable behaviors and lifestyles that can affect people's general health and well-being, including fertility [38]. As a result of changeable lifestyle factors, there are reports of deteriorating reproductive health indicators from the last 5 to 6 decades from different parts of the world, particularly in developed countries [39]. The role of lifestyle factors in the etiology of infertility has drawn increasing attention among researchers. Numerous studies have provided evidence of the relationship between lifestyle behaviors and infertility in women. Factors such as high-fat diet, smoking, alcohol and caffeine consumption, physical activity, high-risk sexual behaviors, anxiety, and radiation exposure affect women's fertility [40, 41]. The results of a study on couples who tried to conceive naturally during 12 months reported that the chances of fertility in couples with four factors, three factors, two factors, and one adverse lifestyle factor were 38%, 52%, 62%, and 71%, respectively. Likewise, in couples with no adverse factors in their lifestyle, the chance of fertility was 83% [42].

The third factor extracted in this study is the chance of conception (CHC). The chance of conception in women decreases with increasing age, and risks such as miscarriage and less success in vitro fertilization (IVF) threaten their reproductive ability [43]. The results of a brief report by Halleran et al. (2022) about fertility knowledge among women trying to conceive without medical intervention showed that almost 70% of women did not know that the week before ovulation is associated with the highest chance of conception [44]. On the other hand, the success rate after one cycle (IVF) for women under 35 has been reported to be around 30%. Still, this percentage drops significantly after the mother's age of 35, and there is almost no chance of a live birth for women over 45 who wish to use IVF [45]. Because of the inaccessibility and high cost of ART, women trying to get pregnant must be equipped with the necessary knowledge to maximize their chances of conceiving without the need for medical intervention.

Finally, ovarian reserve and preservation (ORP) was identified as the fourth factor in this study. One of the determining factors of a woman's fertility potential is ovarian reserve, which is affected by age, genetics, and environmental factors. Ovarian reserve in women depends on the size of the ovarian follicular pool and the quality of the eggs. As the age of a woman increases, the ovarian reserve, as well as the quantity and quality of eggs, decreases. As a result, the reproductive performance of women decreases over time [46]. The results of Azhar et al.'s (2015) study on Knowledge of ovarian reserve and reproductive choices revealed that if the test results indicate a decrease in ovarian reserve, 48% of women try to have children sooner, 21% choose oocyte cryopreservation, 7% try to find their life partner sooner, 7% go for adoption and 3% choose embryo cryopreservation. Only 14% of women do not actively pursue treatment or change their lifestyle [47]. Consequently, increasing awareness about women's ovarian reserve inspires people to improve their life choices. Meanwhile, healthcare providers, particularly midwives, should inquire about couples' childbearing plans and educate couples who postpone childbearing about the natural pattern of fertility decline.

The factors extracted from the MFKI questionnaire align with the results of some studies conducted in this field. The first factor extracted in this study was environment and reproductive health (ERH). In most of the world, men are the decision-makers of families and significantly influence the decisions related to contraception and STI prevention [48]. However, men's reproductive health, which is considered one of the most important health issues in society, has been less investigated. The concept of reproductive health is not limited only to women. Accordingly, men also have the right to receive appropriate reproductive health services that enable them to have a healthy reproductive life during marriage [49]. Men's reproductive health is an essential element of having children, which is affected by environmental issues in addition to physical diseases. Unfavorable environmental factors can lead to poor sperm quality, a decrease in sperm concentration and sperm motility, and an increase in sperm DNA fragmentation index and mitochondrial dysfunction, all of which lead to infertility in men [50].

The second extracted factor is Lifestyle factors (LFS). Factors related to lifestyle are one of the important reasons for male infertility today. The results of the studies revealed that sperm quality is essentially affected by things such as diet, nicotine addiction, alcohol consumption, and exposure to high radiation [51]. The results of numerous studies show that some of the causes of male infertility are the result of an unfavorable lifestyle. Diets high in processed meats, red meat, simple carbohydrates, and high-fat dairy products are associated with both sperm quality and pregnancy outcomes [52]. It has been found that obesity, heavy smoking, use of androgenic steroids, alcohol dependence, and drug use can impair sperm count, motility, morphology, and sexual function [53]. Endocrine-disrupting chemicals such as bisphenol A and phthalates, heavy metals, pesticides, air pollution, and electromagnetic frequencies from computers and cell phones are also associated with reduced sperm concentration, volume, motility, and survival [54].

The third factor recognized in this study was sperm quality (SQ). Sperm quality is the key indicator of men's fertility and the main key to a healthy birth in couples. Regarding male fertility, a meta-regression analysis reported a significant decline in total sperm count worldwide between 1973 and 2011 [55]. The results of numerous studies in men reveal that physical activity is related to improving the quality of sperm and seminal fluid [56]. However, intense physical activity may adversely affect seminal fluid parameters. The probable mechanism of this effect is assumed to be systemic inflammation caused by intense exercise or heat of the scrotum [57]. On the other hand, chronic and acute infections of the genital tract can also be common causes of infertility in men. Mumps virus (MuV) infection often causes orchitis and can lead to testicular atrophy and infertility. MuV induces an immune response through TLR2 and RIG-I signaling, increasing proinflammatory cytokines and chemokines [58]. Chromosomal abnormalities are another factor affecting the quality of men's sperm. Genetic factors are detected in 15% of male infertility cases and can be classified into two groups, chromosomal abnormalities and single gene mutations. About 14% of men with azoospermia and 2% with oligospermia have chromosomal abnormalities [59].

## Strength and limitation

Among the strengths of the current study is examining the psychometric properties of MFKI and FFKI for the first time in Iran. The design of two distinct and particular tools to measure the women's and men’s knowledge state is one of the strengths of this study. On the other hand, this study pointed to limitations such as not calculating criterion validity due to the lack of a gold standard for measuring couples' fertility knowledge. Likewise, the difficulty in retrieving women's life partners are the current study's limitations. Consequently, in the future, more quantitative studies in different cultures, considering larger sample sizes, are recommended to measure couples' knowledge of fertility.

## Conclusion

The findings of the present study indicates that the Persian version of MFKI and FFKI has acceptable psychometric properties to measure the khowledge of Iranian couples regarding fertility, which can be used as a screening tool for fertility knowledge by health care professionals and also as a reliable tool in research.

## Data Availability

The datasets used and/or analyzed during the current study are available from the corresponding author upon reasonable request.

## Abbreviations

MFKI: Male Fertility Knowledge Inventories
FFKI: Female Fertility Knowledge Inventories
ERH: Environment and reproductive health
LSF: Lifestyle factors
SQ: Sperm quality
RH: Reproductive health, CHC: Chance of conception
ORP: Ovarian reserve and preservation
CFKS: Cardiff Fertility Knowledge Scale
BMI: Body mass index
WHO: World health organization
DP: dual panel
EFA: Exploratory factor analysis
CFA: Confirmatory factor analysis
ICC: Intra-class Correlation Coefficient
SD: standard deviation
CI: Confidence interval
CVI: Content validity index
CVR: Content validity ratio
df: degree of freedom
KMO: Kaiser-Meyer-Olkin
RMSEA: Root mean squared error of approximation
CFI: Comparative fit index
TLI: Tucker–Lewis index
SRMR: Standardized root mean squared residual
